# The Patient Perspective on the Impact of Tenosynovial Giant Cell Tumors on Daily Living: Crowdsourcing Study on Physical Function and Quality of Life

**DOI:** 10.2196/ijmr.9325

**Published:** 2018-02-23

**Authors:** Monique Josephine Mastboom, Rosa Planje, Michiel Adreanus van de Sande

**Affiliations:** ^1^ Department of Orthopedics Leiden University Medical Center University of Leiden Leiden Netherlands

**Keywords:** synovitis, pigmented villonodular, giant cell tumor of tendon sheath, rare diseases, crowdsourcing, social media, patient-reported outcome measures, quality of life, health-related quality of life, social participation, surveys and questionnaires

## Abstract

**Background:**

Tenosynovial giant cell tumor (TGCT) is a rare, benign lesion affecting the synovial lining of joints, bursae, and tendon sheaths. It is generally characterized as a locally aggressive and often recurring tumor. A distinction is made between localized- and diffuse-type. The impact of TGCT on daily living is currently ill-described.

**Objective:**

The aim of this crowdsourcing study was to evaluate the impact of TGCT on physical function, daily activities, societal participation (work, sports, and hobbies), and overall quality of life from a patient perspective. The secondary aim was to define risk factors for deteriorated outcome in TGCT.

**Methods:**

Members of the largest known TGCT Facebook community, PVNS is Pants!!, were invited to an e-survey, partially consisting of validated questionnaires, for 6 months. To confirm disease presence and TGCT-type, patients were requested to share histological or radiological proof of TGCT. Unpaired t tests and chi-square tests were used to compare groups with and without proof and to define risk factors for deteriorated outcome.

**Results:**

Three hundred thirty-seven questionnaires, originating from 30 countries, were completed. Median age at diagnosis was 33 (interquartile range [IQR]=25-42) years, majority was female (79.8% [269/337]), diffuse TGCT (70.3% [237/337]), and affected lower extremities (knee 70.9% [239/337] and hip 9.5% [32/337]). In 299 lower-extremity TGCT patients (32.4% [97/299]) with disease confirmation, recurrence rate was 36% and 69.5% in localized and diffuse type, respectively. For both types, pain and swelling decreased after treatment; in contrast, stiffness and range of motion worsened. Patients were limited in their employment (localized 13% [8/61]; diffuse 11.0% [21/191]) and sport-activities (localized 58% [40/69]; diffuse 63.9% [147/230]). Compared with general US population, all patients showed lower Patient-Reported Outcomes Measurements Information System-Physical Function (PROMIS-PF), Short Form-12 (SF-12), and EuroQoL 5 Dimensions 5 Levels (EQ5D-5L) scores, considered clinically relevant, according to estimated minimal important difference (MID). Diffuse versus localized type scored almost 0.5 standard deviation lower for PROMIS-PF (*P*<.001) and demonstrated a utility score of 5% lower for EQ-5D-5L (*P*=.03). In localized TGCT, recurrent disease and ≥2 surgeries negatively influenced scores of Visual Analog Scale (VAS)-pain/stiffness, SF-12, and EQ-5D-5L (*P*<.05). In diffuse type, recurrence resulted in lower score for VAS, PROMIS-PF, SF-12, and EQ-5D-5L (*P*<.05). In both types, patients with treatment ≤1year had significantly lower SF-12.

**Conclusions:**

TGCT has a major impact on daily living in a relatively young and working population. Patients with diffuse type, recurrent disease, and ≥2 surgeries represent lowest functional and quality of life outcomes. Physicians should be aware that TGCT patients frequently continue to experience declined health-related quality of life and physical function and often remain limited in daily life, even after treatment(s).

## Introduction

Tenosynovial giant cell tumor (TGCT), previously pigmented villonodular synovitis (PVNS), is a rare, proliferative neoplasm affecting the synovial lining of joints, bursae, and tendons sheaths. According to growth pattern, a radiological distinction is made between a well-circumscribed lesion (localized type) and a locally more aggressive lesion (diffuse type) [1,2]. The incidence rate reveals its rarity: for localized type (excluding digits), 10.2 per million person-years and for diffuse type, 4.1 per million person-years. TGCT is a monoarticular disease, concerning large joints, typically about the knee: 46% in localized-type and 64% to 75% in diffuse-type. Male-female ratio is about 1:1.5 for both types, with a median age at the time of TGCT diagnosis of 30 to 50 years [1-3]. Most common initial symptoms are pain, stiffness, and swelling. Additional symptoms might be limited range of motion, instability, giving way, and locking complaints [4]. Due to these unspecific signs and the rarity of the disease, patients frequently experience a delay of years in diagnosis [3,5,6]. To treat these symptoms, current treatment of choice is surgical excision, either by arthroscopic or open synovectomy [7]. After surgical resection, high recurrence rates are known, with the localized type up to 50% and the diffuse type up to 92% [6].

Once TGCT is diagnosed, a high health care burden is identified with a significant increase in health care costs, ambulatory expenses, and physical therapy [8]. In describing treatment benefits and standard oncologic end points, patient-reported outcome instruments are increasingly used. Visual Analog Scale (VAS) for worst pain-stiffness and Patient-Reported Outcomes Measurement Information System-Physical Function (PROMIS-PF) questionnaires were identified as most valuable measures for TGCT symptoms in a relatively small TGCT patient cohort (n=22) [4].

The impact of TGCT symptoms following surgery(s) and recurrences on daily living, sports, and work activities is currently ill-described. Although TGCT is not considered lethal, this tumor is hypothesized to have major impact on daily living. Especially diffuse disease is notorious for its negative influence on both local recurrence risk and functional outcome [9].

Use of an e-survey is a unique possibility to reach a large elusive TGCT population and to globally evaluate impact of TGCT on patients’ daily life. This crowdsourcing study evaluates effect of TGCT on physical function, daily activities, societal participation (work, sports, and hobbies), and overall quality of life from a patient perspective. Secondary aim is to define risk factors for deteriorated outcome in TGCT.

## Methods

### Study Design

This cross-sectional crowdsourcing study was performed at Leiden University Medical Center, Leiden, The Netherlands, in accordance with good clinical practice [the Declaration of Helsinki (2000)]. This study was conducted from December 2016 until end of May 2017 (6 months), using the largest known online TGCT community on Facebook, PVNS is Pants!!, to gather participants for the Web-based questionnaire. The study was conducted conforming to the Checklist for Reporting Results of Internet E-Surveys (CHERRIES), the checklist focusing on Web-based surveys [10] (Multimedia Appendix 1). NetQuestionnaire (NetQ) was used to complete the TGCT questionnaire. NetQ is a professional Web-survey software, approved for (bio)medical research and supported by the Leiden University Medical Center (LUMC). Respondents were able to review and change their answers before submitting. 

### Patients and Recruitment

Members of PVNS is Pants!! were requested to participate in our international crowdsourcing study “Evaluation of Tenosynovial Giant Cell Tumor on daily living” (Multimedia Appendix 2). At the time of writing (December, 2016), this closed Facebook community contained 2179 members. A patient-friendly TGCT-research-related message was posted in the Facebook community every 4 weeks to encourage TGCT patients to complete the questionnaire. Additional study updates and easily understandable information on TGCT were posted on the page of a newly designed TGCT study Facebook account [11].

All members of the Facebook community had access to the questionnaire. Solely patients with TGCT diagnosis were requested to participate in this study. To achieve a higher level of evidence, confirmation of TGCT (histological or radiological) was requested after completing the questionnaire. Sending (anonymized) medical reports to our protected email account was highly desirable but left to the discretion of the participant.

Members of Facebook community PVNS is Pants!! have been notified that (research-minded) doctors are members of this closed Facebook community for several years. Participation in this study was voluntary, and no incentives were offered. Informed consent was given by completing the survey. This study was approved by the Institutional Review Board (CME) from our institution (registration number P16.232, December 5, 2016).

Unique site visitors were determined by Internet protocol (IP) addresses. When duplicate entries were detected, the most recent one was included in the analyses. All password-protected documents were only accessible to TGCT researchers and saved on the secured departmental drive of our hospital. Data of participants were anonymized when medical proof was received or when the participant did not respond to our third request for medical confirmation. To ascertain TGCT diagnosis and TGCT type, all medical reports were verified by 2 TGCT researchers (MJLM, RP). When in disagreement, medical reports were checked by the senior orthopedic surgeon (MAJS) for final conclusion.

### Questionnaire

On the very active Facebook community PVNS is Pants!!, several patient-initiated questionnaires and polls were performed, for instance, about treatments, coping strategies, daily limitations, and emotional struggles. Members expressed their desire for studies regarding these topics, since the majority of TGCT studies concern physical function and recurrent disease as outcome parameters. Therefore, a Web-based questionnaire, using mostly validated questionnaires, was composed to describe impact of TGCT on health-related outcome and daily living from a patient perspective. A prerequisite was that the questionnaire would be relevant for the heterogeneous TGCT population: for different large joints, different ages, males or females, localized or diffuse type, and for patients at different treatment stages.

To assess relevance and completeness of our questionnaire, a pilot test with the composed questionnaire was performed. One dedicated orthopedic oncologic surgeon (MAJS), 2 medical doctors (MJLM, RP), and 5 TGCT patients in our outpatient clinic, all fluent in written and spoken English language, tested the e-survey. Validated questionnaires were used as published by the owners. After the pilot test, a few nonvalidated questions were added or rephrased (Multimedia Appendix 3).

Nonvalidated questions concerned patient and tumor characteristics, medical history, TGCT symptoms, performed treatments, recurrences, employment status, sports, and number of visits to general practitioner and orthopedic surgeon. The majority of questions had a multiple-choice character, including a *not applicable* or *other* answer option. The exact number of nonvalidated questions depended on given answers. For instance, patients with an extensive TGCT-related history were asked additional questions on their history, in contrast to the patients awaiting their initial treatment.

Validated questionnaires on physical function and quality of life included: VAS for worst pain and stiffness in the last 24 hours, PROMIS-PF items, Short Form-12 Health Survey (SF-12), and EuroQoL EQ-5D-5L (EQ-5D-5L Descriptive System and EQ-5D-5L VAS). A total of 32 validated questions were included. VAS for pain and stiffness was used to estimate patient’s pain and stiffness intensity for the past 24 hours: *no pain/stiffness at all* (0) and *worst pain/stiffness imaginable* (10).

PROMIS-PF instruments were used to measure self-reported capability of physical activities. In this study, short forms of physical functioning for lower and upper extremity were used with 5 response options: without any difficulty (5), with a little difficulty (4), with some difficulty (3), with much difficulty (2), and unable to do (1). Raw score was calculated by summing up the values of the response to each question and was converted into a T score by the Assessment Center from PROMIS-PF. A mean of a standardized T score of 50 with a standard deviation of 10 reflects the general US population [12].

The SF-12, a generic measure of health status, functioned as a shorter alternative for the SF-36. Number of answer options differed per question. Physical component summary (PCS) score and mental component summary (MCS) score were calculated. Similar to PROMIS-PF, the general US population had a mean of 50 with a standard deviation of 10 [13].

The EQ-5D-5L is one of the most commonly used generic health status measures in the world. Its descriptive system comprises 5 dimensions of health: mobility, self-care, usual activities, pain or discomfort, and anxiety or depression, with the following 5 levels of problems per dimension: no problems (1), slight problems (2), moderate problems (3), severe problems (4), and extreme problems (5) [14]. For each participant, answers per dimension were combined into an EQ-5D-5L health state. This health state was converted into a single index value (so-called utility score) for quality of life, by using the Crosswalk Index Value Calculator version 1.0 from the EuroQoL Group [15]. Utility scores were measured on an ordinal scale of 0 to 1, with 0 indicating death and 1 indicating full health [16]. Crosswalk valuation set for US population was used for all participants, since majority of the patients originated from the United States (42.7% [144/377]). A specific analysis, called sensitivity analysis, was performed using the valuation set for UK population, the second largest patient population (20.2% [68/337]) in this study. Scores calculated with US valuation set were compared with scores obtained by using UK valuation set to assess representativeness of the scores from validated questionnaires [14] (Multimedia Appendix 4).

### Statistical Analysis

NetQ automatically captured questionnaire answers into an SPSS 23 file. Evaluation of TGCT on daily living was mainly descriptive.

Chi-square tests were used to compare patient groups with and without medical proof regarding gender (male vs female), TGCT localization (knee vs other large lower extremity joints [hip, ankle, and foot]), initial surgery (arthroscopy vs [one- or two-staged] open synovectomy), recurrence (yes vs no), total number of surgeries (1 surgery vs ≥2 surgeries), and time since last treatment for TGCT (≤1 year ago vs >1 year ago) (Multimedia Appendix 5).

Independent *t* tests were used to compare the mean age at the time of diagnosis and continuous scores of validated questionnaires. All reported *P* values were two-tailed. Statistical significance level was defined at *P*<.05.

Effect size, as a quantitative measure of the strength of a phenomenon, was calculated for both PROMIS-PF and SF-12 scores in localized- and diffuse-type patients, compared with general US population score. Effect size, or Cohen *d*, is the ratio of difference between two means divided by the standard deviation, expressed in standard deviation units. An effect size between 0.2 and 0.5 is considered small, 0.5 and 0.8 medium, and above 0.8 large [17].

The minimal important difference (MID), a quality of life measure, represents the smallest difference or change beyond statistical significance in an outcome measure score that would be considered clinically relevant by the value patients place on change. MID for EQ-5D-5L Index Scores is estimated between .037 and .069, based on the simulation-based instrument-defined MID estimates [18]. MID for PROMIS-PF was determined by Yost et al in advanced-stage cancer patients [19]. Differences in T scores between 4.0 and 6.0 were considered clinical relevant. MID for SF-12 PCS and MCS scores were calculated by Díaz-Arribas et al in >450 patients with low back pain and were stated at >3.29 for PCS and >3.77 for MCS [20].

## Results

**Figure 1:**
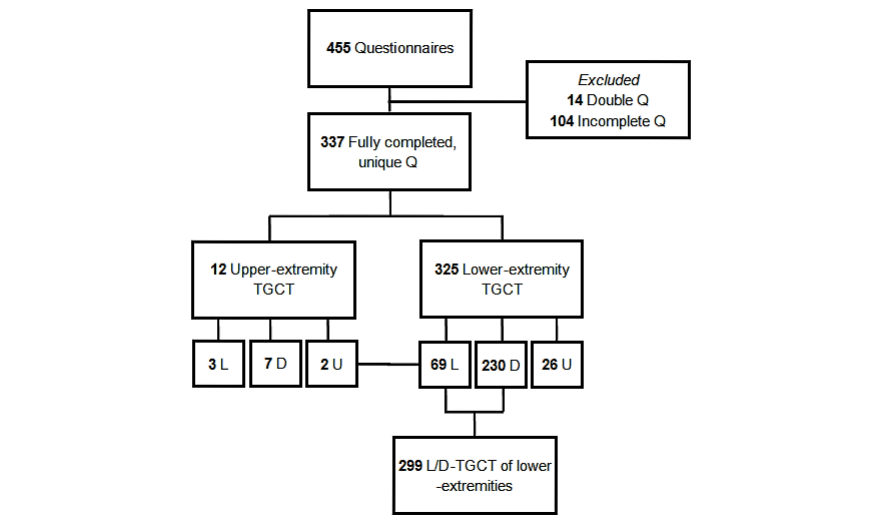
Flowchart Tenosynovial Giant Cell Tumor (TGCT) questionnaire. Q: Questionnaires; L: Localized-TGCT; D: Diffuse-TGCT; U: Unknown-type TGCT.

The TGCT questionnaire was initiated by 445 participants within a time frame of 6 months. For the present analysis, only fully completed, unique questionnaires (337) were included (Figure 1). The majority of incomplete questionnaires were early dropouts with a great lack of information and therefore unsuitable for analysis.

Most patients were female (79.8% [269/337]) and median age at diagnosis was 33 (interquartile range [IQR]=25-42) years. Patients originated from 30 different countries (United States: 42.7% [144/337]; United Kingdom: 20.2% [68/337]; and the Netherlands: 12.8% [43/337]). TGCT was typically located in lower extremities: knee (70.9% [239/337]), hip (9.5% [32/337]), ankle (11.0% [37/337]), and foot (3.0% [10/337]). Diffuse TGCT was diagnosed in 237 of 337 (70.3%) patients (Table 1). According to few TGCT patients with TGCT located in the upper extremity, 12 out of 337 patients (3.6%) were excluded for further analyses. Additionally, 26 out of 337 lower-extremity patients (7.7%) with unknown TGCT type were also excluded (Figure 1). Questionnaires of 299 lower-extremity patients with localized or diffuse TGCT were analyzed.

### Disease Confirmation

Confirmation of TGCT was sent by 32.4% (97/299) of lower-extremity participants. In 81% (78/97) TGCT type was in concordance with questionnaire answer, in 16/97 (16%) medical reports TGCT type did not match the answer and was therefore adjusted according to the report and 3/97 (3%) patients answered TGCT type unknown and TGCT type was added in consistence with the report.

No important differences between patients with and without medical proof were detected (Multimedia Appendix 5), neither for localized or diffuse type separately. Therefore, patients with medical proof were considered representative for the entire study population and additional analyses were performed for the entire patient group.

### Medical History and Tenosynovial Giant Cell Tumor Symptoms

5/69 (7%) and 29/230 (12.6%) in localized- and diffuse-type patients, respectively, had an autoimmune disease, mostly diabetes mellitus type I, Hashimoto, psoriasis, and thyroid disease. In all, 22/69 (32%) of localized-type and 70/230 (30.4%) of diffuse-type patients experienced a trauma at TGCT-affected joint, before diagnosis; sports injuries or fall incidents leading to a sprain or rupture. In all, 5/69 (7%) and 12/230 (5.2%) of patients in localized and diffuse TGCT had surgery of the affected joint before TGCT diagnosis, respectively, for example, meniscus or anterior cruciate ligament (ACL) reconstructions. In all, 6/230 (2.6%) of diffuse-type participants experienced both trauma and surgery before TGCT diagnosis.

**Table 1:** Patient and tumor characteristics (n=337).

Characteristic		Value
Age at time of questionnaire (years), median (IQR^a^)		41 (32-50)
Age at time of TGCT diagnosis (years), median (IQR)		33 (25-42)
Total, N (%)		337 (100)
**Gender, n (%)**		
	Male	68 (20.2)
	Female	269 (79.8)
**Country of residence, n (%)**		
	United States of America	144 (42.7)
	United Kingdom	68 (20.2)
	The Netherlands	43 (12.8)
	Australia	22 (6.5)
	Canada	14 (4.2)
	Other	46 (13.6)
**TGCT^b^localization, n (%)**		
	Knee	239 (70.9)
	Hip	32 (9.5)
	Ankle	37 (11.0)
	Foot	10 (3.0)
	Shoulder	4 (1.2)
	Elbow	6 (1.8)
	Wrist	2 (0.6)
	Other^c^	7 (2.1)
**TGCT type, n (%)**		
	Localized	72 (21.4)
	Diffuse	237 (70.3)
	Unknown	28 (8.3)

^a^IQR: interquartile range (25-75%).

^b^TGCT: tenosynovial giant cell tumor.

^c^Other included multiple TGCT locations (all in lower extremity).

**Table 2:** Initial and current symptoms for localized and diffuse tenosynovial giant cell tumor (TGCT) (n=337).

TGCT^a^-related symptom	Localized TGCT (n=69)		Diffuse TGCT (n=230)	
	Initial, n (%)	Current, n (%)	Initial, n (%)	Current, n (%)
Pain	57 (83)	47 (68)	186 (80.9)	170 (73.9)
Swelling	53 (77)	29 (42)	190 (82.6)	139 (60.4)
Stiffness	38 (55)	41 (59)	128 (55.7)	148 (64.3)
Limited range of motion	38 (55)	29 (42)	140 (60.9)	149 (64.8)

^a^TGCT: tenosynovial giant cell tumor.

Majority of patients (92.6% [277/299]) were treated for TGCT. For both types, pain and swelling improved compared with initial situation. After treatment, more patients reported stiffness and limited range of motion (Table 2). A minority of the patients (<6%) currently experienced additional symptoms, including instability, buckling, hyperextension and/or hypermobility, clicking or locking or popping of joint, numbness, electric shocks, tingling, dull ache, heat of the affected joint, or hematoma.

### Treatment(s)

Most performed initial surgery was arthroscopic synovectomy (57% [38/67] localized, 53.8% [113/210] diffuse) and open synovectomy, one- or two-staged (39% [26/67] localized, 42.9% [90/210] diffuse). In all, 5/67 (7%) localized-type and 53/210 (25.2%) diffuse-type patients had adjuvant therapies after initial surgery, mainly radiotherapy and 90-Yttrium. In all, 24/67 (36%) of localized type had recurrent disease after 1.5 (range 1-6) years, in contrast to 146/210 (69.5%) of diffuse type after 2.2 (range 1-23) years (Table 3). Additional surgery was performed in 23/67 (34%) of localized type and 125/210 (59.5%) of diffuse type, predominantly open synovectomy (one- or two-staged).

### Impact of Tenosynovial Giant Cell Tumor on Daily Life

Due to TGCT, 8/61 (13%) and 21/191 (11.0%) of working population in localized and diffuse TGCT, respectively, was currently not able to (fully) perform their employment. Of these patients, 4/8 (50%) localized patients and 17/21 (81%) diffuse patients had recurrent disease. Majority of patients, 40/69 (58%) of localized and 147/230 (63.9%) of diffuse type, were unable to perform sport activities. In these patients, recurrent disease presented in 15/40 (38%) of localized type and 94/147 (63.9%) of diffuse type. Disease burden was estimated by mean number of visits to general practitioner (5.6 [range 1-50] visits for localized type, 7.1 [range 1-60] visits for diffuse type), and orthopedic surgeon (8.3 [range 1-97] visits for localized type, 11.9 [range 1-100] visits for diffuse type).

Results of validated questionnaires are shown in Table 4 (localized vs diffuse type), Table 5 (localized type), and Table 6 (diffuse type). Results with positive association are described in the text.

### Worst Pain and Stiffness in Last 24 Hours: Visual Analog Scale Score

For localized type, best VAS pain score was 2.76 and VAS stiffness score was 2.80. In diffuse type, best scores for pain and stiffness were 3.04 and 3.08, respectively. Patients with recurrence of TGCT had deteriorated VAS score for pain and stiffness (*P=*.01 localized type and *P*<.001 diffuse type). In localized type, patients with ≥2 surgeries had higher VAS score for pain (*P=*.02) and stiffness (*P*=.01).

### Patient-Reported Outcomes Measurements Information System-Physical Function: T Score

All TGCT patients had clinically relevant impaired T scores (44.5 and 41.3 for localized and diffuse type, respectively) compared with the general US population (T score of 50). Corresponding effect size was medium for localized type (*d*=0.55) and large for diffuse type (*d*=0.87). When comparing both types, diffuse-type patients scored lower (*P*<.001). In localized type, female patients scored lower (*P*=.04). Diffuse-type recurrent patients had decreased scores (*P=*.02).

**Table 3:** Treatment characteristics of 277 treated tenosynovial giant cell tumor (TGCT) patients.

Treatment		Localized-TGCT^a^ (n=67)	Diffuse TGCT (n=210)
**Initial surgery, n (%)**			
	Arthroscopic synovectomy	38 (57)	113 (53.8)
	Open synovectomy (one- or two-staged)	26 (39)	90 (42.9)
	Combined arthroscopic/open synovectomy	3 (4)	0 (0.0)
	Total joint replacement/(tumor) prosthesis	0 (0)	5 (2.4)
	Amputation	0 (0)	2 (1.0)
**Adjuvant therapy, n (%)**		5 (7)	53 (25.2)
	Radiotherapy	4 (6)	18 (8.6)
	90-Yttrium	1 (1)	14 (6.7)
	Systemic	0 (0)	15 (7.1)
	Other^b^	0 (0)	6 (2.9)
Recurrent disease, n (%)		24 (36)	146 (69.5)
**Additional surgery, n (%)**		23 (34)	125 (59.5)
	Arthroscopic synovectomy	7 (10)	32 (15.2)
	Open synovectomy (one- or two-staged)	10 (15)	74 (35.2)
	Combined arthroscopic/open synovectomy	1 (1)	4 (1.9)
	Total joint replacement/(tumor) prosthesis	2 (3)	12 (5.7)
	Amputation	3 (4)	3 (1.4)

^a^TGCT: tenosynovial giant cell tumor.

^b^Other adjuvant therapies were cryosurgery, burning tools, steroid injections, or combination of multiple adjuvant therapies.

**Table 4:** Risk factor comparison of 69 localized versus 230 diffuse tenosynovial giant cell tumor (TGCT) of lower extremities.

TGCT^a^ -type	Worst pain VAS^b^ score, 0 best score and 10 worst score		Worst stiffness VAS score, 0 best score and 10 worst score		PROMIS-PF^c^ T score, mean 50 (SD 10), MID^d^ 4.0–6.0			SF-12^e^, PCS^f^ score, mean 50 (SD 10), MID >3.29			SF-12, MCS^g^ score, mean 50 (SD 10), MID >3.77			EQ-5D-5L DS^h^ utility score, 0 death and 1 full health, MID .037–.069	
	Mean	*P* value	Mean	*P* value	Mean	*d*^i^	*P* value	Mean	*d*	*P* value	Mean	*d*	*P* value	Mean	*P* value
Localized	3.36	.24	3.46	.14	44.5	0.55	*<.001*	40.5	0.95	.08	47.5	0.25	.40	0.76	*.03*
Diffuse	3.79		4.01		41.3	0.87		38.1	1.19		46.3	0.38		0.72	

^a^TGCT: tenosynovial giant cell tumor.

^b^VAS: Visual Analog Scale.

^c^PROMIS-PF: Patient-Reported Outcomes Measurement Information System-Physical Function.

^d^MID: minimal important difference represents the smallest difference or change beyond statistical significance in an outcome measure score that would be considered important by the value patients place on change ref [18-20].

^e^SF: Short-Form.

^f^PCS: physical component summary.

^g^MCS: mental component summary.

^h^DS: descriptive system.

^i^*d*: Cohen *d* or effect size, ratio of difference between 2 means divided by the standard deviation.

### Short Form-12 Health Survey: Physical and Mental Component Summary Score

In comparison with general US population (score of 50), both types had impaired PCS (40.5 localized and 38.1 diffuse type) and MSC scores (47.5 localized and 46.3 diffuse type). In all patients in all compared groups, PCS score was clinically relevant declined, in contrast to MCS score which did not transcend the MID threshold in majority of patient groups. A large effect size was calculated for mean PCS scores (0.95 and 1.19 for localized and diffuse type, respectively) and a medium effect size (0.25 and 0.38 for localized and diffuse type, respectively) for MCS scores. In localized type, higher number of surgeries (≥2) affected PCS score negatively (*P=*.03). Localized- and diffuse-type patients who underwent treatment for TGCT ≤1 year ago, showed lower PCS score (*P=*.04 localized, *P=*.01 diffuse). In diffuse type, female patients demonstrated a decreased MCS score (*P*=.04), as well as patients with recurrence of TGCT (*P*=.04).

### EuroQoL 5 Dimensions 5 Levels Health Questionnaire: Index Value

All patients, in all groups (Tables 4-6), presented declined EQ5D-5L utility scores compared with full health (1), and all scores transcended MID threshold. Overall, utility score was lower in diffuse patients compared with localized patients (*P*=.03). In localized type, participants with recurrence of TGCT and ≥2 surgeries scored lower (*P*=.01 and *P*=.02, respectively). Similarly, diffuse patients with recurrence had decreased scores (*P*=.02). Median health question VAS score was 75 (IQR 65-85) for localized and 75 (IQR 56.5-85) for diffuse type. No differences between scores calculated with US and UK valuation sets were detected in sensitivity analysis (Multimedia Appendix 4).

**Table 5:** Risk factor comparison of 69 localized tenosynovial giant cell tumor (TGCT) of lower extremities.

Risk-factors		Worst pain VAS^a^ score, 0 best score and 10 worst score		Worst stiffness VAS score, 0 best score and 10 worst score		PROMIS-PF^b^ T score, mean 50 (SD 10), MID^c^ 4.0-6.0		SF-12^d^, PCS^e^ score, mean 50 (SD 10), MID >3.29		SF-12, MCS^f^ score, mean 50 (SD 10), MID >3.77		EQ-5D-5L DS^g^ utility score, 0 death and 1 full health, MID .037-.069	
		Score	*P* value	Score	*P* value	Score	*P* value	Score	*P* value	Score	*P* value	Score	*P* value
**Gender**													
	Male (n=14)	2.93	0.53	3.29	.79	48.5	*.04*	43.3	.23	49.4	.42	.81	.18
	Female (n=55)	3.47		3.51		43.5		39.8		47.0		.75	
**Age of diagnosis**													
	<35 years (n=36)	3.36	.997	3.39	.82	44.1	.63	40.1	.74	45.4	.07	.76	.95
	≥35 years (n=33)	3.36		3.55		45.0		40.9		49.8		.77	
**TGCT localization**													
	Knee (n=53)	3.04	.08	3.13	.07	44.2	.57	41.0	.43	47.2	.63	.77	.41
	Hip, ankle, foot, other (n=16)	4.44		4.56		45.5		38.8		48.6		.74	
**Initial surgery**													
	Arthroscopy (n=38)	3.45	.58	3.26	.63	44.2	.52	40.7	.96	47.2	.73	.76	.92
	Open surgery^h^ (n=26)	3.04		3.62		45.6		40.8		48.0		.77	
**Recurrence**													
	Yes (n=24)	4.50	*.01*	4.71	*.01*	42.8	.20	38.3	.17	45.7	.27	.70	*.01*
	No (n=45)	2.76		2.80		45.4		41.7		48.5		.80	
**Total no. of surgeries**													
	1 surgery (n=44)	2.77	*.02*	2.86	*.01*	45.4	.20	42.2	*.03*	48.4	.19	.79	*.02*
	≥2 surgeries (n=23)	4.48		4.65		42.6		36.7		45.0		.71	
**Last treatment for TGCT**													
	≤1 year ago (n=31)	3.77	.26	3.81	.38	42.4	.06	37.6	*.04*	46.0	.34	.74	.29
	>1 year ago (n=36)	3.00		3.19		46.1		42.6		48.3		.78	

^a^VAS: Visual Analog Scale.

^b^PROMIS-PF: Patient-Reported Outcomes Measurement Information System-Physical Function.

^c^MID: minimal important difference represents the smallest difference or change beyond statistical significance in an outcome measure score that would be considered important by the value patients place on change ref [18-20].

^d^SF: Short-Form.

^e^PCS: physical component summary.

^f^MCS: mental component summary.

^g^DS: descriptive system.

^h^One- or two staged open synovectomy.

**Table 6:** Risk factor comparison of 230 diffuse tenosynovial giant cell tumor (TGCT) of lower extremities.

Risk factors		Worst pain VAS^a^ score, 0 best score and 10 worst score		Worst stiffness VAS score, 0 best score and 10 worst score		PROMIS-PF^b^ T score, mean 50 (SD 10), MID^c^ 4.0-6.0		SF-12^d^, PCS^e^ score, mean 50 (SD 10), MID >3.29		SF-12, MCS^f^ score, mean 50 (SD 10), MID >3.77		EQ-5D-5L DS^g^ utility score, 0 death and 1 full health, MID .037-.069	
		Score	*P* value	Score	*P* value	Score	*P* value	Score	*P* value	Score	*P* value	Score	*P* value
**Gender**													
	Male (n=51)	3.63	.70	4.13	.71	42.2	.23	39.9	.11	49.0	*.04*	.75	.17
	Female (n=179)	3.84		3.98		41.0		37.5		45.6		.71	
**Age of diagnosis**													
	<35 years (n=119)	3.70	.58	3.74	.09	42.1	.07	39.2	.07	46.9	.38	.73	.22
	≥35 years (n=109)	3.89		4.32		40.4		36.8		45.6		.71	
**TGCT localization**													
	Knee (n=170)	3.78	.93	3.92	.07	41.6	.29	38.1	.99	46.3	.98	.73	.40
	Hip, ankle, foot, other (n=60)	3.82		4.55		40.5		38.1		46.3		.71	
**Initial surgery**													
	Arthroscopy (n=113)	3.93	.82	4.19	.64	41.6	.66	38.3	.86	45.6	.64	.73	.25
	Open surgery^h^ (n=190)	3.84		4.01		41.2		38.0		46.4		.70	
**Recurrence**													
	Yes (n=146)	4.23	*<.001*	4.55	*<.001*	40.5	*.02*	37.7	.49	45.1	*.04*	.70	*.02*
	No (n=84)	3.04		3.08		42.7		38.7		48.2		.75	
**Total no. of surgeries**													
	1 surgery (n=86)	3.79	.69	3.74	.09	42.0	.20	38.7	.48	46.1	.89	.73	.44
	≥2 surgeries (n=124)	3.94		4.38		40.8		37.7		46.3		.71	
**Last treatment for TGCT**													
	≤1 year (n=72)	4.10	.37	4.35	.37	40.4	.17	35.4	*.01*	45.6	.59	.70	.17
	>1 year ago (n=138)	3.76		4.00		41.8		39.5		46.5		.73	

^a^VAS: Visual Analog Scale.

^b^PROMIS-PF: Patient-Reported Outcomes Measurement Information System-Physical Function.

^c^MID: minimal important difference represents the smallest difference or change beyond statistical significance in an outcome measure score that would be considered important by the value patients place on change ref [18-20].

^d^SF: Short-Form.

^e^PCS: physical component summary.

^f^MCS: mental component summary.

^g^DS: descriptive system.

^h^One- or two staged open synovectomy.

## Discussion

### Principal Findings

The name of the largest online community of patients with TGCT, PVNS is pants!!, suggests impact on quality of life. One of the community members motivated the name: “Pants is British slang for crap or garbage.” To date, it is unknown what the effect of TGCT on daily living is. A questionnaire was composed in consultation with TGCT patients to determine functional, socioeconomic, and health burden for TGCT patients. We intended to evaluate TGCT in the real world and concluded that TGCTs have a large impact on daily living, with declined health-related quality of life and limitations in daily activities, sports, work, and hobbies: especially the diffuse type of lower extremities and recurrent disease including multiple surgeries.

### Limitations

The most important limitation to this study is selection bias. By using crowdsourcing to gather data, it is likely to have a higher number of patients with severe or recurrent diseases [21]. Consequently, when extrapolating these results to generally described populations of TGCT patients in literature, care should be taken not to overestimate the decreased physical function and additional socioeconomic limitations. TGCT usually affects young adults. Since younger patients are more likely to be on the World Wide Web, and our included patient population had a median age of 33 (25-42) years at time of diagnosis, also in concordance with the WHO classification [1,2] and Mastboom et al [3], we considered our participants representative for the heterogeneous disease TGCT. Additionally, the CHERRIES was completed. This checklist provides an understanding of the sample (self-)selection and its possible differences from a representative sample [10] (Multimedia Appendix 1). An additional limitation to this study is that patients in different stages of different treatments were included. To assess comparability within study population, we compared patients who had treatment less than a year ago with performed treatment over a year ago. No positive associations were discovered, except for the SF-12 PCS score in both types. This underlines the postoperative limitations during the first year of follow-up after treatment. As we set out to evaluate impact of TGCT on daily living in the real world heterogeneous TGCT population, the inclusion of patients in different treatment stages matched intention of our study. Furthermore, a known disadvantage of quality of life questionnaires (eg, SF-12) is the generalizability of the questions. Impaired overall quality of life could be attributed to TGCT but also to additional physical abnormalities or psychological problems. Also questionnaires may be completed by patients that have been ill-informed on their disease. In all, 28 patients filled out *unknown type of TGCT*, and 16% of patients who confirmed TGCT with medical proof filled out localized TGCT instead of diffuse TGCT or vice versa. Undeniably, differentiating in localized and diffuse TGCT is challenging even for (un)specialized physicians. The relatively high recurrence rate in this study could also be reflected by unawareness of disease specifics. Recurrence rates in our study were 36% and 70% for localized and diffuse type, compared with on average 4% to 6% (up to a maximum of 50%) and 14% to 40% (up to a maximum of 92%) according to van der Heijden et al [6], respectively. It is conceivable that residual disease or clinical symptoms were filled out as recurrent disease.

The use of self-reported questionnaires harbors the risk of incorrectly answered questions. One could argue that all patients should have been analyzed together, not subdividing into localized and diffuse type. However, differences between two types are major, and therefore separate analyses were necessary for a realistic view of impact of TGCT on daily living.

### Crowdsourcing

The presumed definition of crowdsourcing is the practice of obtaining services, ideas, or content by collecting contributions from a comprehensive group from an online community rather than from traditional data suppliers [9]. However, the exact definition for crowdsourcing remains controversial, as 40 definitions originating from 32 unique articles, published between 2006 and 2011, were described by Estellés-Arolas [22]. It is therefore challenging to well define crowdsourcing coherently. After analyses of the 40 (sometimes contrasting) definitions, 8 characteristics common to any given crowdsourcing initiative were found: the crowd, the task at hand, the recompense obtained, the crowdsourcer or initiator of the crowdsourcing activity, what is obtained by them following the crowdsourcing process, the type of process, the call to participate, and the medium. First, in our study, the crowd is presented by patients with TGCT (preferably confirmed by medical reports). Second, the task at hand is completing a questionnaire about the effect of TGCT on daily living. Third, participating in this study was voluntary, therefore no recompenses were offered. Fourth, the initiators of this study are members of the Facebook group PVNS is Pants!! accompanied with the executors, known as the authors of this paper. Fifth, the researchers and subsequently the participants and TGCT patients gain more knowledge on the impact of TGCT on daily living. Sixth, the type of process is an evaluation process, aiming to evaluate effect of TGCT on daily living. Seventh, all patients with TGCT, fluent in English language, were invited to complete the questionnaire. Lastly, the medium Facebook was used to broadcast the questionnaire.

Facebook is the best applicable social network site for survey research, because it is continuously growing, internationally known and exceeds 2 billion users globally (June 2017). The Facebook community PVNS is Pants!!, created in 2009, is the largest TGCT online support group and mainly consists citizens of the United States. On this very active, closed Facebook community, patients are daily updating experiences on their disease, ask for advice from fellow TGCT patients, and comment on other posts to provide their knowledge or sympathy. By actively posting and commenting on research proposals, patients expressed their willingness to participate in research on TGCT. From these posts, we learned that adequate patient information on TGCT is lacking. Our crowdsourcing study stimulated patients’ involvement in research and was an opportunity to align research questions with the public’s interest [23,24]. TGCT is a rare disease and time to definitive diagnosis is prolonged due to unspecific symptoms and unfamiliarity of the disease [5]. A challenge in studying a rare disease is the lack of big data. Crowdsourcing is an effective and low-cost alternative to traditional methods of participant recruitment due to the possibility to reach large groups of individuals in a relatively short time frame [25]. Van der Heijden et al [9] concluded that crowdsourcing is a promising way for evaluation of rare diseases. Czajka et al [21] used crowdsourcing to efficiently recruit a global cohort and is the largest study on patients with multiple hereditary exostoses. Crosier et al [26] used Facebook to recruit patients with auditory hallucinations; within 6 weeks, over 250 patients had completed this survey. Pohlig et al [27] concluded that enrollment of patients in prospective studies is time-consuming and could be facilitated by use of crowdsourcing.

To obtain a higher level of scientific value, patients were requested for medical proof to ascertain TGCT diagnosis. To our knowledge, no other crowdsourcing studies considered disease confirmation. Patient data and outcome for validated questionnaires were comparable for patients with and without medical proof. Patients were not uniformly diagnosed and treated as they originated from 30 different countries globally. Neither was distinguished between treatment in peripheral or tertiary referral centers. Nevertheless, we consider our study group a reflection of the current worldwide situation and believe that declined impact on daily living is clinically relevant for all patients. In contrast to malignant diseases, survival rates are not of interest for TGCT with its benign character. According to high recurrence rates, quality of life (prior and after treatment) is essential to evaluate.

### Patient-Reported Outcome Measures

Patient reported outcome measures (PROMs) are increasingly used in health policy, patient-centered care, and shared clinical decision making [28]. In the era of personalized medicine, patient involvement is increasing in shared decision making for different treatment strategies with functional outcome and quality of life. In our study, members of the largest online TGCT community were involved in establishing the questionnaire *Evaluation of TGCT on daily living*.

Functional outcome and health-related quality of life are only spars reported for TGCT. Four studies have reported on standardized PROMS [4,9,29,30]. Currently, validated PROMS for TGCT patients do not exist. In accordance with Gelhorn et al [4], VAS for worst pain and stiffness and PROMIS-PF questionnaires were used. Conform van der Heijden et al [9,29] and Verspoor et al [30], the SF-12, a quality of life questionnaire, was included, known as the shorter version of the SF-36. One study identified a high health care burden with a significant increase in health care costs, ambulatory costs, and physical therapy in 9328 TGCT patients [8].

In benign diseases, including TGCT, death is not an outcome variable. Besides tumor reduction, critical endpoint measures are clinical relevance and impact of treatment. Currently, clinical TGCT studies lack specific and validated PROMs to document treatment-induced symptomatic, functional, and economic (back to work) improvement [31]. To obtain an impression of physical function and quality of life in TGCT patients, participants in our study were requested to complete different validated questionnaires. In our experience, PROMIS-PF was most useful in determining these functional factors. To minimize the multitude of questions and include the most important components for clinical TGCT studies, we would propose a combination of PROMIS-PF and a short quality of life questionnaire, for instance EQ5D5L, in clinical practice.

### Risk Factors for Deteriorated Outcome

Risk factors for deteriorated outcome in our study were diffuse-type TGCT, recurrent disease, and ≥2 surgeries performed. This is in concordance with current literature on risk factors for a high recurrence rate. According to the necessity of mutilating surgeries to treat recurrences, we considered risk factors for recurrent disease comparable to risk factors for deteriorated outcome.

Higher recurrence rate in diffuse TGCT compared with localized TGCT is exuberant described [2,5-7,30,32-34]. Bruns et al [34] described 173 patients treated in 10 orthopedic departments in Germany and Austria and reported higher recurrence rates in institutions treating less than 20 cases for TGCT, in diffuse disease, in the hip joint and after arthroscopy. Schwartz et al [35] described 99 patients with TGCT in the knee, hip, elbow, or shoulder. They concluded that localization in the knee, previous surgical procedures, and incomplete synovectomy were related significantly to higher number of subsequent recurrences. On the basis of current literature and to investigate possible risk factors for recurrent disease thoroughly, gender, age at time of diagnosis, TGCT localization, initial surgery, presence of recurrence, total number of surgeries, and time since last treatment for TGCT, were compared.

### Conclusions

TGCTs have major impact on daily living in a relatively young, working population (median age at diagnosis, 33 years). Majority of symptoms improve after treatment, however, symptoms remain in about half of the TGCT patients; especially in patients with diffuse type, recurrent disease, and ≥2 surgeries. The high recurrence rate in diffuse TGCT results in clinically important deteriorated outcome in physical function and health-related quality of life. In preventing recurrent disease, and its deteriorated outcome, an extensive mutilating surgery might be necessary. Physicians should be aware that TGCT patients frequently experience symptoms and limitations in daily life and societal participation (work, sports, and hobbies), even after treatment(s). We deem it important for future research to evaluate treatment, including its effectiveness on improving quality of daily living. With this study, we hope to increase knowledge on TGCT among treating physicians, highlight the importance of quality of life, and to offer research-based information to patients.

## Appendix

Multimedia Appendix 1 Data reporting guidelines, checklist for reporting results of internet E-Surveys (CHERRIES).

Multimedia Appendix 2 Facebook introduction, invitation to complete questionnaire and thank you message.

Multimedia Appendix 3 Questions e-survey "Evaluation of TGCT on daily living".

Multimedia Appendix 4 Supplementary material—sensitivity analysis.

Multimedia Appendix 5 Comparing patients with medical proof to patients without medical proof.

## Abbreviations

ACL: anterior cruciate ligament
CHERRIES: Checklist for Reporting Results of Internet E-surveys
CME: Commissie Medische Ethiek (Institutional Review Board from the LUMC)
DS: descriptive system
EQ-5D-5L: EuroQoL 5 Dimensions 5 Levels
IP: Internet protocol
IQR: interquartile range
JMIR: Journal of Medical Internet Research
LUMC: Leiden University Medical Center
MCS: mental component summary
MID: minimal important difference
NetQ: NetQuestionnaire
PCS: physical component summary
PROMIS-PF: Patient-Reported Outcomes Measurements Information System-Physical Function
PROMS: Patient Reported Outcome Measures
PVNS: pigmented villonodular synovitis
SF-12: Short Form-12 Health Survey
SPSS: Statistical Package for the Social Sciences
TGCT: tenosynovial giant cell tumor
VAS: Visual Analog Scale
