# Supplementary material for: The Patient Perspective on the Impact of Tenosynovial Giant Cell Tumors on Daily Living: Crowdsourcing Study on Physical Function and Quality of Life
Source: Interact J Med Res. 2018 Feb 23;7(1):e4. doi: 10.2196/ijmr.9325 (PMC5845102; doi:10.2196/ijmr.9325)
Supplement: Multimedia Appendix 1 [file ijmr_v7i1e4_app1.pdf]

Appendix I Data reporting guidelines, checklist for reporting results of internet E-Surveys (CHERRIES)

Item category	*Checklist item*	*This study*
**Design**	Describe survey design	Target population was 2179 TGCT patients enrolled in closed Facebook group ‘‘PVNS is pants!!’’. [December, 2016]
**IRB approval and informed consent process**	IRB approval	This study was approved by the Institutional review board (CME) from our institution [registration number P16.232, December 5th, 2016].
	Informed consent	Informed consent was given by completing the e-survey.
	Data protection	Password protected documents were only accessible to researchers and saved on the secured departmental drive of our hospital. The study folder was stored on the departmental network drive (‘R-schijf’), data security was ensured by LUMC ICT protection. Data of participants were anonymized when medical proof was received or when the participant did not respond to our third request for medical reports.
**Development and pre-testing**	Development and testing	Survey developed via NetQuestionnair, a professional survey software, supported by Leiden University Medical Center (LUMC).
**Recruitment process and description of the sample having access to the questionnaire**	Open survey versus closed survey	Open survey on closed Facebook group.
	Contact mode	Contact with participants through the Facebook community, link to the survey was provided here.
	Advertising the survey	The e-survey was announced on the Facebook community wall with a first notice of the study, and repeated requests monthly [Appendix II]. Similar information regarding this study was posted at own TGCT-Facebook page.
**Survey administration**	Web/E-mail	E-survey was posted on a website (i.e. closed Facebook group ‘PVNS is pants!!’).<br>Responses were automatically captured into an SPSS 23 file, only accessible to TGCT-researchers.
	Context	A TGCT-based information and support community on Facebook, with mostly patients, but also family members and researchers. Information and personal experiences concerning TGCT is shared; its (surgical) treatment, recovery duration, tips and tricks.
	Mandatory/voluntary	Voluntary survey.
	Incentives	No incentives were offered.
	Time/date	December 2016–June 2017.
	Randomization of items or questionnaires	No randomization.
	Adaptive questioning	Adaptive questioning for non-validated questionnaires
	Number of items	Appendix III, a maximum of 60 questions, depending on certain answers (32 validated questions).
	Number of screens (pages)	1 Webpage per survey, if applicable.
	Completeness check	Completeness checks were done through JavaScript, not applicable options were included.
**Response rates**	Review step	Respondents were able to review and change their answers while completing the e-survey.

	Unique site visitor	Unique site visitors were determined by IP addresses and email addresses.
	View rate (ratio of unique survey visitors/unique site visitors)	If assumed that all members of ‘PVNS is pants!!’ viewed questionnaire: 570/2179 = 26%
	Participation rate (ratio of unique visitors who agreed to participate/unique first survey page visitors)	455/570 = 80% (570 patients opened link to the survey, 455 patients started the questionnaire)
	Completion rate (ratio of users who finished the survey/users who agreed to participate)	352/455 = 77% (352 completed questionnaires)<br>337/455 = 76% (337 completed, unique questionnaires)
**Preventing multiple entries from the same individual**	Cookies used	No cookies were used.
	IP check	Duplicate database entries having the same user ID/IP address were eliminated before analyses; the most recent entries were included.
	Log file analysis	See above.
	Registration	N.A.
**Analysis**	Handling of incomplete questionnaires	Only completed questionnaires were analyzed.
	Questionnaires submitted with an atypical timestamp	N.A.
	Statistical correction	No difference was detected between patients with and without medical TGCT proof for demographics and validated questionnaires.
