# Supplementary material for: The Patient Perspective on the Impact of Tenosynovial Giant Cell Tumors on Daily Living: Crowdsourcing Study on Physical Function and Quality of Life
Source: Interact J Med Res. 2018 Feb 23;7(1):e4. doi: 10.2196/ijmr.9325 (PMC5845102; doi:10.2196/ijmr.9325)
Supplement: Multimedia Appendix 2 [file ijmr_v7i1e4_app2.pdf]

Appendix II Facebook introduction, invitation to complete questionnaire and thank you message

Tgct Study
September 26, 2016

Dear PVNS is pants group,

We are four medical researchers from the Leiden University Medical Center (LUMC), the Netherlands: Michiel Van De Sande, Monique Mastboom, Rosa Planje en Lucianne Remijn. Our study is called: TGCT (PVNS) in the real world. We want to discover the ins and outs of this orphan disease. Of course this is only possible by asking experts: this is why we ask you as TGCT patient to become member of our TGCT Study page.

Previously, our colleague, Lizz van der Heijden, investigated surgical, oncological and functional outcome and quality-of-life (QOL) in patients. We feel it is important to continue study on the rare TGCT and explore the effect of TGCT on daily living.

We would propose to compose a questionnaire. In this way we hope to get a reliable impression about your daily complaints, the extent of limitations in performance of daily activities, repetitive trauma-injury's, medical history, social life and (overall) financial situation.

We would like to know if you would be interested to collaborate in this study! You can find more information about TGCT and our study on our TGCT page, so we would like to invite you to become friends with us (Tgct Study)! In this way you can also give your input e.g. suggestions for questions!

Looking forward to your input in TGCT in the real world!
Lucianne Remijn

Daniel Uittenbogaard and 64 others 58 Comments

Like Comment

Tgct Study shared a link.
December 2, 2016

Dear 'PVNS is pants' members!

Previously, we told you about our plan to start the TGCT study: TGCT in the real world. We are pleased to share good news with you!

The TGCT-questionnaire to explore TGCT in the real world is online since TODAY. Only by asking experts it's possible to discover ins and outs of this orphan disease. We kindly invite you to join the TGCT study by filling out the questionnaire.

The purpose of our study is to get a reliable impression of your daily complaints, the extent of limitations in daily activities, repetitive trauma-injury's, medical history, social life and (overall) financial situation.

The following link will give you access to the questionnaire:
https://orthopedie.netqsurvey.lumc.nl/nq.cfm...

We are looking forward to hear your answers. Many thanks in advance!

Kind regards from the Netherlands, on behalf of the TGCT research team,

Michiel van de Sande
Monique Mastboom
Rosa Planje
Lucianne Remijn

Evaluation of Tenosynovial Giant Cell Tumour (TGCT) on daily living

Welcome to the questionnaire "Evaluation of TGCT on daily living". This questionnaire is composed to gather information on Tenosynovial Giant Cell Tumour (TGCT), previously called Pigmented VilloNodular Synovitis (PVNS); a rare benign but possibly locally aggressive disease. Please only continue wit...

ORTHOPEDIE.NETQSURVEY.LUMC.NL

Lucianne Remijn, Rosa Planje and 30 others 28 Comments

Like Comment Share

Tgct Study is at LUMC.
June 6 · Sassenheim

Dear 'PVNS is pants members',

A BIG THANKYOU to all of you who have participated in our international, crowdsourcing study by filling out the questionnaire 'Evaluation of TGCT on daily life'. We hope to find answers to your initiated questions and will start analyses next month. This month, we hope to receive more confirmations of TGCT, in order to increase purity and scientific value to this study. The questionnaire is now closed, it is not possible to complete it anymore.

We are so pleased that many of you have participated in our study! In total, we have received 338 completed questionnaires. We really appreciate all your input, enthusiasm and willingness to help.

A big shout out to Sheila Rae in particular, for promoting our study, answering our questions regarding the group PVNS is pants and helping us gathering information about this rare disease.

The other administrators of PNVS is pants have been very supporting too by thinking along and accepting our posts in this closed support-group!

We hope to acquire more knowledge and understanding of this orphan disease in the future! Of course, we will share the results of our study with you.

We kindly request you to send a copy of your pathology, radiology or physician report to our protected email tgctresearch@lumc.nl when you have completed our questionnaire. Thank you very much in advance!

Kind regards on behalf of the TGCT Study Team,

Michiel van de Sande
Monique Mastboom
Rosa Planje
Lucianne Remijn

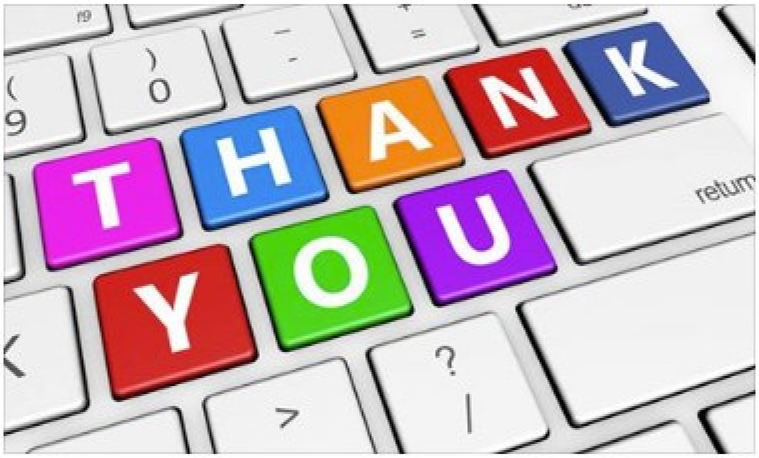

Lucianne Remijn, Rosa Planje and 13 others 1 Comment

Like Comment
