# Supplementary material for: The Patient Perspective on the Impact of Tenosynovial Giant Cell Tumors on Daily Living: Crowdsourcing Study on Physical Function and Quality of Life
Source: Interact J Med Res. 2018 Feb 23;7(1):e4. doi: 10.2196/ijmr.9325 (PMC5845102; doi:10.2196/ijmr.9325)
Supplement: Multimedia Appendix 3 [file ijmr_v7i1e4_app3.pdf]

Appendix III Questions e-survey ‘Evaluation of TGCT on daily living’

Non-validated questions

	Question	Answer options
**1**	What is your email address?	Open question, *optional*
**2**	What is your gender?	Male<br>Female
**3**	What is your year of birth?	Open question
**4**	In which country do you live?	Open question
**5**	What is your employment status?	Employed<br>Self-employed<br>Unemployed<br>Student<br>Retired<br>Other, specify ______
**6**	Please specify employment status	Full time paid employment<br>Part-time paid employment, due to TGCT<br>Part-time paid employment, due to other reasons<br>No paid employment due to TGCT<br>No paid employment due to TGCT but receiving disability aid paid by the government<br>No paid employment due to other reasons<br>Retired due to TGCT<br>Retired due to other reasons
**7**	Do you have any auto-immune diseases (e.g. rheumatoid arthritis, SLE, Diabetes Mellitus type I?)	No<br>Yes, please specify disease ______
**8**	Do you have any auto-immunes disease s in the family?	No<br>Yes, please specify disease ______
**9**	At which age were you first diagnosed with TGCT?	Open question
**10**	Where is TGCT localized?	Knee<br>Hip<br>Ankle<br>Foot<br>Shoulder<br>Elbow<br>Wrist<br>Hand<br>Other, please specify ______
**11**	Did you experience trauma and/or surgery at location of TGCT prior to diagnosis (e.g. fracture, (repetitive) sprain, rupture)?	No<br>Yes, I experienced trauma, but no previous surgery at location of TGCT [please specify trauma] ______<br>Yes, I experienced surgery, but no trauma at location of TGCT [please specify surgery] ______<br>Yes, I experienced trauma at location of TGCT prior to diagnosis and underwent surgery at location of TGCT [please specify trauma and surgery] ______
**12**	Which type of TGCT is diagnosed?	Localized (giant cell tumor tendon sheath)<br>Diffuse (previous PVNS)<br>Soft tissue<br>Unknown
**13**	What were your <u>initial</u> symptoms? [*more answer-options possible]*	None<br>Pain<br>Stiffness<br>Swelling<br>Limited range of motion<br>Other, specify ______
**14**	What are your <u>current</u> symptoms? [*more answer-options possible]*	None<br>Pain<br>Stiffness<br>Swelling<br>Limited range of motion<br>Other, specify ______
**15**	How many GP (General Practitioner) <u>visits</u> due to TGCT did you have? *[estimation] [please put 99 when unknown]*	Open question

16	How many Orthopedic surgeon <u>visits</u> due to TGCT did you have? *[estimation] [please put 99 when unknown]*	Open question
17	Which initial surgery did you have?	I did not have any surgery<br>Arthroscopy *(small surgical incisions)*<br>Open synovectomy (one-stage) (*large surgical incisions)*<br>Combined/two-staged synovectomy (*at least two surgical incisions)*<br>Total joint replacement/(tumor)prosthesis<br>Amputation<br>Other, specify ______
18	How many <u>years</u> ago did the initial surgery take place? *[If surgery took place less than a year ago, fill in 0]*	Open question
19	Which adjuvant treatment did you have?	Nothing<br>Radiotherapy<br>90-Yttrium<br>Systemic<br>Cryosurgery<br>Other, specify ______
20	How many <u>weeks</u> in rehabilitation/physical therapy did you have? *[If you did not have any rehabilitation/physical therapy, fill in 0]*	Open question
21	Which additional/second surgery did you have?	I did not have any surgery<br>Arthroscopy *(small surgical incisions)*<br>Open synovectomy (one-stage) (*large surgical incisions)*<br>Combined/two-staged synovectomy (*at least two surgical incisions)*<br>Total joint replacement/(tumor)prosthesis<br>Amputation<br>Other, specify ______
22	How many <u>years</u> ago did additional/second surgery take place? *[If surgery took place less than a year ago, fill in 0]*	Open question
23	What is the total number of surgeries you have had?	1 surgery<br>2 surgeries<br>3 surgeries<br>4 surgeries<br>5 surgeries<br>5+ surgeries
24	Did you experience any recurrences of TGCT?	No<br>Yes
25	After how many <u>years</u> did recurrence of TGCT take place? *[If recurrence took place less than a year ago, fill in 0]*	Open question
26	Did you have a joint replacement?	No<br>Yes
27	How long was the in hospital <u>follow-up</u> period (controls in order to monitor TGCT)? *[years] [If follow-up was less than a year ago, fill in 0]*	Open question
28	Were you TGCT free at final follow-up?	I did not have my final follow-up yet<br>No<br>Yes
29	Are you currently able to perform sports?	No<br>Yes, once a week<br>Yes, two times a week<br>Yes, three times a week<br>Yes, four times or more per week
30	Which sport(s) do/did you perform? *[If you do not perform sports, please put X]*	Open question
31	Space for questions/suggestions/notes from the participant	Open question, *optional*

Validated questionnaire I: EQ-5D-5L Health Questionnaire

Under each heading, please tick the ONE box that best describes your health TODAY.

Question	Answer options
Mobility	I have no problems in walking about<br>I have slight problems in walking about<br>I have moderate problems in walking about<br>I have severe problems in walking about<br>I am unable to walk about
Self-care	I have no problems washing or dressing myself<br>I have slight problems washing or dressing myself<br>I have moderate problems washing or dressing myself<br>I have severe problems washing or dressing myself<br>I am unable to wash or dress myself
Usual activities (e.g. work, study, housework, family or leisure activities)	I have no problems doing my usual activities<br>I have slight problems doing my usual activities<br>I have moderate problems doing my usual activities<br>I have severe problems doing my usual activities<br>I am unable to do my usual activities
Pain/discomfort	I have no pain or discomfort<br>I have slight pain or discomfort<br>I have moderate pain or discomfort<br>I have severe pain or discomfort<br>I have extreme pain or discomfort
Anxiety/depression	I am not anxious or depressed<br>I am slightly anxious or depressed<br>I am moderately anxious or depressed<br>I am severely anxious or depressed<br>I am extremely anxious or depressed
We would like to know how good or bad your health is TODAY. This scale is numbered from 0 to 100;<br>• 100 means the best health you can imagine;<br>• 0 means the worst health you can imagine.<br>Please write the number for your health today.	Open question (0-100)

Validated questionnaire II: Visual Analogue Scale (VAS) for worst pain/stiffness in last 24 hours

Question	Answer options
Worst pain over the last 24 hours<br><br>Please rate your pain by choosing the one number that best describes your pain at its worst in the last 24 hours;<br>• 0 means no pain;<br>• 10 means pain as bad as you can imagine.	0-10
Worst stiffness over the last 24 hours<br><br>Please rate your pain by choosing the one number that best describes your stiffness at its worst in the last 24 hours;<br>• 0 means no stiffness;<br>• 10 means stiffness as bad as you can imagine.	0-10

Validated questionnaire III: Patient-Reported Outcomes Measurement Information System-Physical Functioning (PROMIS-PF); upper- and lower extremity short forms

Upper extremity

Please respond to each item by marking one box per row.

Question	Answer options
PFB34: Are you able to change a light bulb overhead?	Without any difficulty<br>With a little difficulty<br>With some difficulty<br>With much difficulty<br>Unable to do
PFA16r1: Are you able to dress yourself, including tying shoelaces and buttoning up your clothes?	Without any difficulty<br>With a little difficulty<br>With some difficulty<br>With much difficulty<br>Unable to do
PFB54: Does your health now limit you in going OUTSIDE the home, for example to shop or visit a doctor's office?	Without any difficulty<br>With a little difficulty<br>With some difficulty<br>With much difficulty<br>Unable to do
PFA4: Does your health now limit you in doing heavy work around the house like scrubbing floors, or lifting or moving heavy furniture?	Without any difficulty<br>With a little difficulty<br>With some difficulty<br>With much difficulty<br>Unable to do
PFA12: Are you able to push open a heavy door?	Without any difficulty<br>With a little difficulty<br>With some difficulty<br>With much difficulty<br>Unable to do
PFB28r1: Are you able to lift 10 pounds (5 kg) above your shoulder?	Without any difficulty<br>With a little difficulty<br>With some difficulty<br>With much difficulty<br>Unable to do
PFA14r1: Are you able to carry a heavy object (over 10 pounds/5 kg)?	Without any difficulty<br>With a little difficulty<br>With some difficulty<br>With much difficulty<br>Unable to do
PFB1: Does your health now limit you in doing moderate work around the house like vacuuming, sweeping floors or carrying in groceries?	Without any difficulty<br>With a little difficulty<br>With some difficulty<br>With much difficulty<br>Unable to do
PFA5: Does your health now limit you in lifting of carrying groceries?	Without any difficulty<br>With a little difficulty<br>With some difficulty<br>With much difficulty<br>Unable to do
PFA42: Are you able to carry a laundry basket up a flight of stairs?	Without any difficulty<br>With a little difficulty<br>With some difficulty<br>With much difficulty<br>Unable to do
PFA13: Are you able to exercise for an hour?	Without any difficulty<br>With a little difficulty<br>With some difficulty<br>With much difficulty<br>Unable to do

Lower extremity

Please respond to each item by marking one box per row.

Question	Answer options
PFA23: Are you able to go for a walk of at least 15 minutes?	Without any difficulty<br>With a little difficulty

	With some difficulty<br>With much difficulty<br>Unable to do
PFA16r1: Are you able to dress yourself, including tying shoelaces and buttoning up your clothes?	Without any difficulty<br>With a little difficulty<br>With some difficulty<br>With much difficulty<br>Unable to do
PFB54: Does your health now limit you in going OUTSIDE the home, for example to shop or visit a doctor's office?	Without any difficulty<br>With a little difficulty<br>With some difficulty<br>With much difficulty<br>Unable to do
PFA4: Does your health now limit you in doing heavy work around the house like scrubbing floors, or lifting or moving heavy furniture?	Without any difficulty<br>With a little difficulty<br>With some difficulty<br>With much difficulty<br>Unable to do
PFA12: Are you able to push open a heavy door?	Without any difficulty<br>With a little difficulty<br>With some difficulty<br>With much difficulty<br>Unable to do
PFA14r1: Are you able to carry a heavy object (over 10 pounds/5 kg)?	Without any difficulty<br>With a little difficulty<br>With some difficulty<br>With much difficulty<br>Unable to do
PFB1: Does your health now limit you in doing moderate work around the house like vacuuming, sweeping floors or carrying in groceries?	Without any difficulty<br>With a little difficulty<br>With some difficulty<br>With much difficulty<br>Unable to do
PFA5: Does your health now limit you in lifting of carrying groceries?	Without any difficulty<br>With a little difficulty<br>With some difficulty<br>With much difficulty<br>Unable to do
PFA21: Are you able to go up and down stairs at a normal pace?	Without any difficulty<br>With a little difficulty<br>With some difficulty<br>With much difficulty<br>Unable to do
PFA42: Are you able to carry a laundry basket up a flight of stairs?	Without any difficulty<br>With a little difficulty<br>With some difficulty<br>With much difficulty<br>Unable to do
PFA10: Are you able to stand for one hour?	Without any difficulty<br>With a little difficulty<br>With some difficulty<br>With much difficulty<br>Unable to do
PFA3: Does your health now limit you in bending, kneeling or stooping?	Without any difficulty<br>With a little difficulty<br>With some difficulty<br>With much difficulty<br>Unable to do
PFA13: Are you able to exercise for an hour?	Without any difficulty<br>With a little difficulty<br>With some difficulty<br>With much difficulty<br>Unable to do

Validated questionnaire IV: SF-12

This survey asks for your views about your health. This information will help keep track of how you feel and how well you are able to do your usual activities. Answer each question by choosing just ONE answer. If you are unsure how to answer a question, please give the best answer you can.

Question	Answer options
In general, would you say your health is ...	Excellent<br>Very good<br>Good<br>Fair<br>Poor
**Health and daily activities**<br>**The following questions are about activities you might do during a typical day. Does your health limit you in these activities? If so, how much?**<br><br>**Moderate activities, such as moving a table, pushing a vacuum cleaner, bowling or playing golf?**	Yes, limited a lot<br>Yes, limited a little<br>No, not limited at all
**Health and daily activities**<br>**The following questions are about activities you might do during a typical day. Does your health limit you in these activities? If so, how much?**<br><br>**Climbing several flights of stairs?**	Yes, limited a lot<br>Yes, limited a little<br>No, not limited at all
**Physical health**<br>**During the past 4 weeks, have you had any of the following problems with your work or other regular daily activities as a result of your physical health?**<br><br>**Accomplished less than you would like.**	No<br>Yes
**Physical health**<br>**During the past 4 weeks, have you had any of the following problems with your work or other regular daily activities as a result of your physical health?**<br><br>**Were limited in the kind of work or other activities.**	No<br>Yes
**Emotional health**<br>**Accomplished less than you would like.**	No<br>Yes
**Emotional health**<br>**Didn't do work or other activities as carefully as usual.**	No<br>Yes
During the past 4 weeks, how much did pain interfere with your normal work (including both work outside the home and housework	Not at all<br>A little bit<br>Moderately<br>Quite a bit<br>Extremely
**Feelings**<br>**Have you felt calm and peaceful?**	All of the time<br>Most of the time<br>A good bit of the time<br>Some of the time<br>A little of the time<br>None of the time
**Feelings**<br>**Did you have a lot of energy?**	All of the time<br>Most of the time<br>A good bit of the time<br>Some of the time<br>A little of the time<br>None of the time
**Feelings**<br>**Have you felt downhearted and blue?**	All of the time<br>Most of the time<br>A good bit of the time<br>Some of the time<br>A little of the time<br>None of the time
**During the past 4 weeks, how much of the time has your physical health or emotional problems interfered with your social activities (like visiting friends, relatives, etc.)?**	All of the time<br>Most of the time<br>Some of the time<br>A little of the time<br>None of the time
