# Supplementary material for: The Patient Perspective on the Impact of Tenosynovial Giant Cell Tumors on Daily Living: Crowdsourcing Study on Physical Function and Quality of Life
Source: Interact J Med Res. 2018 Feb 23;7(1):e4. doi: 10.2196/ijmr.9325 (PMC5845102; doi:10.2196/ijmr.9325)
Supplement: Multimedia Appendix 4 [file ijmr_v7i1e4_app4.pdf]

Supplementary material

Sensitivity analysis for EQ-5D-5L Utility score using USA and UK valuation sets

		USA valuation set<br><br>Score	USA valuation set<br><br>*P*	UK valuation set<br><br>Score	UK valuation set<br><br>*P*
**Localized-TGCT (n 69)**	**Gender**<br>Male | Female<br>(n 14|55)	0.81 | 0.75	.18	0.75 | 0.67	.23
	**Current age**<br><40 years | ≥40 years<br>(n 33|36)	0.77 | 0.77	.99	0.69 | 0.68	.89
	**TGCT localization**<br>Knee | hip, ankle, foot<br>(n 53|16)	0.77 | 0.74	.41	0.71 | 0.60	.12
	**Initial surgery (n 64)**<br>Arthroscopy | open synovectomy<br>(n 38|26)	0.76 | 0.77	.92	0.69 | 0.69	.97
	**Recurrence**<br>Yes| no<br>(n 24|45)	0.70 | 0.80	**<u>.01</u>**	0.59 | 0.74	**<u>.01</u>**
	**Total number of surgeries (n 67)**<br>1 surgery | ≥2 surgeries<br>(n 44|23)	0.79 | 0.71	**<u>.02</u>**	0.73 | 0.60	**<u>.02</u>**
	**Last treatment for TGCT**<br>≤1 year | >1 year ago<br>(n 31|36)	0.74 | 0.78	.29	0.66 | 0.71	.38
**Diffuse-TGCT (n 230)**	**Gender**<br>Male | Female<br>(n 51|179)	0.75 | 0.71	.17	0.66 | 0.61	.16
	**Current age**<br><40 years | ≥40 years<br>(n 105|125)	0.72 | 0.72	.89	0.62 | 0.63	.77
	**TGCT localization**<br>Knee | hip, ankle, foot<br>(n 170|60)	0.73 | 0.71	.40	0.63 | 0.59	.33
	**Initial surgery (n 203)**<br>Arthroscopy | open synovectomy<br>(n 113|90)	0.73 0.70	.25	0.63 | 0.60	.24
	**Recurrence**<br>Yes | no<br>(n 146|84)	0.70 | 0.75	**<u>.02</u>**	0.60 | 0.67	**<u>.01</u>**
	**Total number of surgeries (n 210)**<br>1 surgery | ≥2 surgeries<br>(n 86|124)	0.73 | 0.71	.44	0.63 | 0.61	.45
	**Last treatment for TGCT**<br>≤1 year | >1 year ago<br>(n 72|138)	0.70 | 0.73	.17	0.59 | 0.63	.25
