# Supplementary material for: The Patient Perspective on the Impact of Tenosynovial Giant Cell Tumors on Daily Living: Crowdsourcing Study on Physical Function and Quality of Life
Source: Interact J Med Res. 2018 Feb 23;7(1):e4. doi: 10.2196/ijmr.9325 (PMC5845102; doi:10.2196/ijmr.9325)
Supplement: Multimedia Appendix 5 [file ijmr_v7i1e4_app5.pdf]

Supplementary material I - Comparing patients with medical proof to patients without medical proof

	Proof of TGCT (n 97[a])	No proof of TGCT (n 202[a])	
	**n (%)**	**n (%)**	***P***
Mean age at diagnosis in years (SD)	36.7 (13.2)	33.8 (12.0)	.08
Gender<br>Male | female	24 (25%) | 73 (75%)	41 (20%) | 161 (80%)	.38
TGCT localization<br>Knee | other joints	70 (72%) | 27 (28%)	153 (76%) | 49 (24%)	.49
Initial surgery<br>Arthroscopy | open synovectomy	n 85<br>39 (46%) | 46 (54%)	n 182<br>112 (62%) | 70 (38%)	.05
Recurrence<br>Yes | no	49 (51%) | 48 (49%)	121 (60%) | 81 (40%)	.13
Number of surgeries<br>1 surgery | ≥2 surgeries	n 89<br>48 (54%) | 41 (46%)	n 188<br>82 (44%) | 106 (56%)	.55
	**Mean score**	**Mean score**	***P***
VAS worst pain<br>VAS score	3.6	3.7	.81
VAS worst stiffness<br>VAS score	3.8	4.0	.55
PROMIS-PF<br>T-score	42.3	41.9	.72
SF-12<br>PCS score	37.7	39.1	.32
SF-12<br>MSC score	47.2	46.2	.49
EQ-5D-5L-DS<br>Utility score	0.7	0.7	.72

a, Unless listed otherwise.
